# Causative factors of liver fibrosis in HIV-infected patients. A single center study

**DOI:** 10.1186/s12876-020-01230-1

**Published:** 2020-04-06

**Authors:** Theodoros Androutsakos, Maria Schina, Abraham Pouliakis, Athanasios Kontos, Nikolaos Sipsas, Gregorios Hatzis

**Affiliations:** 1 Department of Pathophysiology, Medical School, National and Kapodistrian University of Athens, Mikras Asias 75, 115 27 Athens, Greece; 2 Liver unit, Euroclinic of Athens, Athens, Greece; 3 Second Department of Pathology, Medical School, National and Kapodistrian University of Athens, Athens, Greece; 4 Infectious Diseases Unit, Laiko General Hospital, Athens, Greece; 5 Department of Pathophysiology, Medical School, National and Kapodistrian University of Athens, Athens, Greece; 6 Department of Pathophysiology, Medical School, National and Kapodistrian University of Athens, Athens, Greece

**Keywords:** HIV infection, Liver fibrosis, CD4/CD8 ratio, HCV/HIV coinfection, Liver stiffness, Transient Elastography

## Abstract

**Background:**

Liver disease is a leading cause of morbidity and mortality among Human Immunodeficiency virus (HIV) infected patients; however no consensus exists on HIV-related risk factors for it. The aim of this study was to identify risk factors for liver fibrosis/cirrhosis in a cohort of Greek HIV-infected patients.

**Methods:**

Patients attending the HIV outpatient clinic of Pathophysiology Department at «Laiko» General Hospital in Athens, Greece, between December 2014 and December 2017 were eligible for inclusion. Inclusion criteria were confirmed HIV infection and age > 18 years. Exclusion criteria were Body-Mass index (BMI) > 40, liver metastases of malignant diseases and concurrent or previous chemotherapy.

Liver stiffness (LS) was measured using Vibration Controlled Transient Elastography (TE) and laboratory tests were acquired in all patients. Patients were classified in 2 groups: those with mild or no fibrosis (equivalent to Metavir score F0-F2) and those with significant fibrosis (equivalent to Metavir score F3-F4).

**Results:**

A total of 187 consecutive patients were included in this study. Median TE value was 5.1 kilopascals (KPa) (range 2.8–26.3), with 92.5% (173/187) of the patients having no/mild fibrosis and 7.4% (14/187) significant fibrosis.

On multivariate logistic regression analysis older patient’s age, abnormal serum aspartate aminotransferase (AST) value, Hepatitis C virus (HCV) infection, alcohol abuse, CD4/CD8 ratio and an increased number of liver related events (LREs) were significantly correlated with liver fibrosis/cirrhosis.

**Conclusions:**

In our cohort of HIV-infected individuals HCV/HIV co-infection, older age, alcohol abuse and CD4/CD8 ratio seem to correlate with fibrogenesis in the liver.

## Background

Human immunodeficiency virus (HIV) infection constitutes a major global public health issue, with 37 million people worldwide, 34 of them being adults, being infected from HIV [1]. After the initiation of antiretroviral treatment (ART) back in 1996, the leading causes of morbidity and mortality among people living with HIV in industrialized countries has shifted from opportunistic infections and AIDS-related neoplasms to non-AIDS defining events, especially cardiovascular and liver diseases [2–5]. In particular, liver-related deaths in HIV infected patients occur 10 times more often in comparison with the general population [6], ranging from 7 to 14% in different studies [7, 8].

Liver conditions complicating HIV infection have also changed during the years. Starting from the early AIDS-period, when the major problem was hepatitis B with or without hepatitis D infection, moving to the 2000s with the rise of hepatitis C virus (HCV) infection and nowadays with the alcoholic and non-alcoholic fatty liver disease, liver involvement has been a major issue in the history of HIV infection [9].

Liver biopsy (LB) remains the gold-standard for diagnosis and staging of liver fibrosis, but its application in everyday clinical practice has many limitations (need for an experienced personnel, high cost, sampling errors, intraobserver variability) and complications (bleeding, pain and rarely death) [10–13]. However, in the last decade, advances in the field of medical technology and imaging allow clinicians to estimate liver status with no invasive procedures. Among them transient elastography (TE) seems to provide the most reliable results with high creditability, being superior to serum biomarkers which sometimes underestimate the magnitude of liver disease [14–18].

Although liver fibrosis is not an uncommon finding in HIV infected patients, there is no agreement over the HIV-related causative risk factors [19–24]. The aim of this study was to identify the main risk factors for liver fibrosis/cirrhosis in a cohort of Greek HIV-infected patients.

## Methods

All consecutive patients attending the HIV outpatient clinic of the Pathophysiology department at «Laiko» General Hospital in Athens, Greece, between December 2014 and December 2017 were eligible for inclusion in this study. Inclusion criteria were confirmed HIV infection and age > 18 years. Exclusion criteria were Body Mass Index (BMI) > 40, malignant disease with metastases in the liver and concurrent or previous chemotherapy. The protocol of the study was reviewed and approved by «Laiko» General Hospital’s ethical committee, informed consent was obtained from each patient included in the study and the study protocol conforms to the ethical guidelines of the Declaration of Helsinki (Edinburgh, 2000).

Liver stiffness (LS) measurement and laboratory work-up (including serum transaminases, cholesterol, high-density lipoproteins (HDL), low-density lipoproteins (LDL), albumin, blood sugar and thyroid stimulating hormone (TSH) serum levels) as well as HIV-infection surrogate markers (CD4 count, CD4/CD8 ratio and HIV viral load) were performed in all patients that met the inclusion criteria. Medical history, including quantity of daily, as well as weekly, alcohol intake, was obtained by interviewing patients and/or through their medical charts. Liver fibrosis was assessed by Vibration Controlled Transient Elastography (TE) using FibroScan (EchoSens, Paris, France) performed at the time of blood collection for laboratory work-up. All TE were performed by the same experienced operator and all blood samples were analyzed in the same laboratory. HIV infection staging has been performed according to Centers for Disease Control and Prevention (CDC) classification system [25]. Duration of HIV infection was defined based on the first positive test for HIV infection by western blotting, while duration of ART regimens was assessed according to patients’ drug prescriptions. A patient was characterized as diabetic if he was on anti-diabetic treatment or if his/her blood sugar level during testing was > 126 mg/dl or > 200 mg/dl for fasting or non-fasting patient respectively and hyperlipidemic if he was on hypolipidemic treatment or if his/her fasting cholesterol levels was > 200 mg/dl and/or LDL was > 130 mg/dl in double check at least 3 months apart [26, 27]. As liver related event (LRE) was defined every event associated with the liver like transaminasemia, acute viral hepatitis, portal vein thrombosis and drug-induced liver injury, with transaminasemia being defined as the existence of serum transaminases levels > 3 x upper limit of normal (ULN), according to reference laboratory values, and hyperbilirubinemia as total serum bilirubin levels > 2 x ULN. As drug induced liver injury was defined every event of transaminasemia or cholestasis attributed to a certain drug taken by the patient and requiring this drug’s discontinuation. As acute viral hepatitis was regarded any event of transaminasemia attributed to a specific virus (t.ex. Epstein-Barr virus (EBV), Hepatitis A virus (HAV), Herpes simplex virus (HSV), Varicella zoster virus (VZV)). As other liver related events were regarded complications affecting the liver but not already mentioned, like acquired immune deficiency syndrome (AIDS) cholangiopathy, liver cancer, autoimmune diseases of the liver or Budd-Chiari syndrome. As alcohol abuse was defined drinking 8 or more drinks per week for women and 15 or more drinks per week for men or binge drinking (defined as 4 drinks for women and 5 drinks for men—in about 2 h) on 5 or more days in 1 month, according to National Institute on Alcohol and Alcoholism guidelines [28].

Patients were considered to have no/mild liver fibrosis (equivalent to Metavir score F0-F2) with LS values < 8.1 Kilopascals (KPa) if they were co-infected with HBV, < 9.4 KPa if they were co-infected with HCV or had alcoholic steatosis and with < 8.4 KPa for patients not included in the above groups. Patients allocated in the ‘significant fibrosis’ group (equivalent to Metavir score F3-F4) were those with LS values higher than the abovementioned ones [14–19, 22].

Demographic, clinical and laboratory characteristics stratified by the severity of liver fibrosis were compared. A correlation analysis estimated possible impacts on liver fibrosis determined by TE. For parameters expressed in numeric form, the Kruskal-Wallis test was applied in order to identify differences between patients in the no/mild and in the significant fibrosis group. To estimate the effect in univariate analysis it was estimated the odds ratio (OR) for the categorical parameters and the epsilon-squared (ε^2^) for the parameters that were measurable in numeric form [29], both with the 95% confidence interval (CI). Note that the effect characterization ε^2^ according to its value is: 0–0.002: minor effect, 0.002–0.04: small effect, 0.04–0.10: intermediate effect and 0.10–0.20: significant effect.

Categorical variables were compared using the Chi-squared test. Multivariate analyses were performed by multiple logistic regression models using backwards elimination; the *p* value to remove a variable from the model was set to be higher than 0.05 and the statistical significance *p* values were set to be < 0.05. In addition, only first order interactions between parameters were allowed. SAS 9.4 for Windows software package (SAS Institute Inc. NC, USA) was used for statistical analysis. Additionally, in univariate analysis, the estimation of the effect and the related 95% CI for the arithmetic characteristics was performed via the R language version 3.6.0.

## Results

This study included a total of 187 HIV-infected patients; 170 (90.9%) were male with 135 (79.4%) of them being men who have sex with men (MSM). The patients’ median age was 46 (range 20–85) years old and the median BMI 24.2 (range 18.3–38). Twenty-three (12.3%) patients were intravenous drug users (IVDU) and 20 (10.7%) patients had a history of alcohol abuse in the last 5 years. Seven patients were diabetic (3 of them on treatment) and 64 (34.2%) had a history of hyperlipidemia (18 of them on treatment). Baseline characteristics, HIV-related medical history, and laboratory results of the patients included in the study are shown in Table 1.

**Table 1:** Patients’ laboratory results

Parameter	Mean ± SD	(Min,Max)	Median (IQR)
Age (years)	46.2 ± 11.5	(20, 85)	46 (38–53)
HIV Duration (months)	110.7 ± 92.8	(1, 381)	84 (37–168)
Duration of treatment (months)	88.0 ± 84.9	(0, 348)	59.5 (16–134)
BMI	24.6 ± 3.5	(18.3, 38.0)	24.2 (22.3–26.4)
ALP (IU/ml)	73.6 ± 25.6	(9, 180)	70.0 (56.0–85.0)
ALT (IU/ml)	32.7 ± 29.8	(8, 286)	24.0 (20–32.5)
AST (Iu/ml)	28.56 ± 22.32	(9, 230)	24.0 (20.0–28.0)
Bil (mg/dl)	0.62 ± 0.59	(0.16, 6.38)	0.49 (0.36–0.67)
gGT (IU/ml)	42.2 ± 46.7	(8, 343)	28.0 (18.0–44.0)
PLTs (k/μl)	213,963 ± 62,336	(65,000, 608,000)	205,000 (176,000-242,000)
TRG (mg/dl)	155.9 ± 114.2	(41, 761)	124.0 (97.0–167.0)
CHOL (mg/dl)	190.1 ± 46.7	(77, 313)	189.0 (154.0–218.0)
CD4 (cells/μL)	697.6 ± 380.7	(10, 1881)	660.0 (412.0–909.0)
CD4 / CD8	0.70 ± 0.42	(0.03, 2.22)	0.65 (0.38–0.95)
Months of CD4 less than 50 cells/μL	1.48 ± 9.2	(0, 120)	0 (0–0)
Months of CD4 less than 200 cells/μL	6.71 ± 20.43	(0, 240)	0 (0–6)
Months of CD4 less than 350 cells/μL	19.22 ± 35.76	(0, 289)	5 (0–20)
Months of VL higher than 50 cop/ml	31.48 ± 43.85	(0, 225)	12 (5–39)
Number of LREs	0.42 ± 0.78	(0, 5)	0 (0–1)
Events of transaminasemia	0.59 ± 1.13	(0, 5)	0 (0–1)

*BMI* Body-mass index, *ALP* Alkaline phosphatase, *ALT* Alanine aminotransferase, *AST* Aspartate aminotransferase, *Bil* Total Bilirubin, *gGT* gamma glutamyl-transferase, *PLTs* platelets, *TRG* triglycerides, *CHOL* cholesterol, *VL* Viral load, *LREs* Liver related events.

The median duration of HIV seropositivity was 84 months (range 1–381) with the commonest way of HIV acquisition being sex (163 patients, 87.1%). The vast majority of the patients (85.6%) were under ART, and the median duration of treatment was 84 months (range 2–348). A total of 37 patients had a traceable viral load when TE was performed, with 27 of them not being under treatment and the other 10 having begun their ART during the last 6 months. One hundred thirteen (60.4%) patients were classified as stage A, 35 (18.7%) stage B and 39 (20.9%) stage C, according to the CDC [25]. LREs occurred in a total of 60 (32.1%) patients, with the commonest event being transaminasemia occurring in 44 patients (73.3%). Full list of LREs is shown in Table 2.

**Table 2:** List of Liver Related Events (LREs) by frequency of occurrence

LRE (***N*** = 60 patients ^a^)	N (%)
Transaminasemia	44 (73.3)
Acute viral hepatitis	3 (5.0)
Portal vein thrombosis	1 (1.7)
Drug-induced liver injury	3 (5.0)
Hyperbiliruminemia	4 (6.7)
Other	12 (20%)

^a^ Note: A single patient may experience more than one different LREs

Ten (5.3%) patients were HBsAg positive, 20 (10.7%) had traceable HCV RNA and 158 (84.5%) were HIV mono-infected. Baseline laboratory results of the included patients are shown in Table 1. Twenty-three (12.3%) and 37 (19.8%) patients had abnormal serum levels of Aspartate aminotransferase (AST) and Alanine aminotransferase (ALT) respectively, 65 (34.8%) patients abnormal serum levels of Gamma-glutamyl transferase (γ-GT), 5 (2.7%) of Alkaline phosphatase (ALP) and 10 (5.3%) patients had high total bilirubin serum levels. Median CD4+ T cell count was 657.5 cells/mm^3^ (range 10–1881). Four (2.1%) patients of the total population had a CD4+ T cell count of less than 50 cells/mm^3^, 17 (9.1%) less than 250 cells/mm^3^, and 38 (20.3%) less than 350 cells/mm^3^.

Median TE value was 5.1 KPa (range 2.8–26.3), with 92.5% (173/187) of the patients being placed in the no/mild fibrosis group (equivalent to Metavir score of F0-F2) and 7.4% (14/187) in the significant fibrosis one (equivalent to Metavir score of F3-F4).

In univariate analysis a higher prevalence of fibrosis was found in patients with higher serum levels of liver injury markers (ALT, AST, γ-GT), older age and increased number of episodes of transaminasemia in medical history. A positive correlation with high LS was also found in univariate analysis among patients with chronic HCV infection, diabetes mellitus, alcohol abuse and clinical AIDS (CDC stage C) (Table 3).

**Table 3:** Univariate analysis of the study parameters that had a positive role for liver fibrosis. Only those that achieved statistical significance are presented

Parameter	Effect estimate ^a^	95% CI	***P*** value
Age (years)	0.023	0.0018–0.067	0.0285
AST (IU/ml)	0.083	0.020–0.17	0.0003
ALT (IU/ml)	0.034	0.0005–0.114	0.0081
γGT (IU/ml)	0.051	0.0057–0.13	0.0012
LRE (Yes vs. No)	4.6	1.5–14.3	0.0051
Number of LREs	0.038	0.0007–0.114	0.0052
Events of transaminasemia	0.060	0.0043–0.16	0.0042
HCV co-infection (Yes vs. No)	4.2	1.2–15.1	0.0178
Alcohol overuse (Yes vs. No)	3.9	1.1–13.9	0.0244
Diabetes Mellitus (Yes vs. No)	5.6	1.0–31.9	0.0307
Clinical AIDS (Stage CDC – C vs. non C)	4.4	1.4–13.4	0.0055

*AST* Aspartate Aminotransferase, *ALT* Alanine Aminotransferase γ-GT: Gamma Glutamyl Transpeptidase, *LRE* Liver related event, *HCV* Hepatitis C, *AIDS* Acquired Immune Deficiency Syndrome.

^a^ Effect is reported as epsilon-square (ε^2^) and odds ratio for the arithmetic and categorical quantities respectively

On the contrary, liver fibrosis stage was not correlated with BMI, gender, sexual orientation or HIV-relevant disease markers (CD4 count, CD4/CD8 ratio, HIV duration, duration of ART and ART-free duration of HIV infection, months of low CD4 count and viral load of more than 50 cop/ml). Also no correlation was found with the drugs used in patients’ ART (see appendix).

On multivariate logistic regression analysis, the model started with all studied variables, including those that had no significant impact on abnormal LS values in the univariate analysis. In a second step, having in mind that laboratory variables (AST, ALT, γ-GT, ALP, total and direct Bilirubin serum levels, platelets’ count, total cholesterol, triglycerides and albumin serum levels) are the result and not the causative factors of liver injury, laboratory values were excluded from the analysis. The variables associated with higher LS values are shown on Table 4. These results show that the variables with most significant influence are those with a significant impact on abnormal LS in univariate analysis, with the only exception of CD4/CD8 ratio, which was not important in univariate analysis. When all variables were used, older age, lower CD4/CD8 ratio, higher AST values and higher number of events of transaminasemia accounted positively to higher probability for abnormal LS values (Table 3). When laboratory results were excluded both HCV co-infection and alcohol abuse appeared as important factors leading to higher LS values, while events of transaminasemia became statistically insignificant (Table 5).

**Table 4:** Multivariate analysis of factors leading to significant liver fibrosis using all study’s parameters

Parameter	Odds Ratio (95% CI)	p
Age	1.1 (1.0–1.2)	0.0023
CD4 / CD8 ratio	0.04 (0.003–0.540)	0.0151
AST	1.08 (1.03–1.13)	0.0026
Events of transaminasemia	2.3 (1.2–4.5)	0.0147

*AST* Aspartate aminotransferase, *CI* Confidence Interval

**Table 5:** Multivariate analysis of factors leading to significant liver fibrosis using all results but biochemical markers

Parameter	Odds Ratio (95% CI)	p
Age	1.1 (1.0–1.2)	0.0056
Alcohol Overuse (Yes)	9.90 (1.9–50)	0.0058
HCV Infection (Yes)	10.6 (1.8–62.5)	0.0091
CD4 / CD8 ratio	0.1 (0.01–0.8)	0.0251

*HCV* Hepatitis C Virus, *CI* Confidence Interval.

## Discussion

HIV-infected patients live longer since the introduction of ART, but they remain a high-risk group for liver and cardiovascular diseases. HIV infection per se, HIV/HBV and HIV/HCV coinfection, ART, diabetes mellitus, BMI and fatty liver infiltration, have all been associated with an increased risk of significant liver damage which leads to liver fibrosis. However, previously published data although largely agree that HIV/HCV co-infection and older age are aggravating factors for significant liver fibrosis, disagree if HIV infection per se or ART drugs can cause chronic liver damage, leading to liver fibrosis [19–24].

To address these questions we conducted a study in a cohort of Greek, HIV-infected, patients, using TE to assess liver fibrosis. Our study using TE demonstrated a rate of no/mild fibrosis (equivalent to Metavir score of F0-F2) in 92.7% of the HIV-infected patients and significant fibrosis (equivalent to Metavir score of F3-F4) in 7.4% of them. Statistical analysis showed that presence of HIV/HCV co-infection, older patient’s age, diabetes mellitus, AST levels and alcohol abuse were associated with the presence of significant liver fibrosis; a higher number of LREs had also a positive effect in it. As far as HIV infection per se is concerned, significant liver fibrosis was correlated with clinical AIDS (CDC staging C) while absolute CD4 count, duration of seropositivity,duration of low CD4 count (both under 350 as well as under 50 cells/mm^3^) and duration of traceable viral load, were not associated with a higher risk of liver fibrosis.

In multivariate analysis, when all parameters were used, older age, AST levels and increased number of events of transaminasemia showed a correlation with significant fibrosis, while higher CD4/CD8 ratio led to lower probability for high LS values. When biochemical values were excluded from multivariate analysis, both alcohol abuse and HIV/HCV co-infection rose as statistically significant fibrosis factors.

In our study univariate analysis indicated that high serum levels of ‘liver damage’ biomarkers γ-GT, AST and ALT correlated with higher LS values. However in multivariate analysis only AST serum levels seemed to correlate well with liver fibrosis. Even though that may seem a bit strange, this finding becomes logical when taking into account that in multivariate analysis HIV/HCV co-infection and alcohol abuse rise as factors leading to high LS. Since both of them lead to higher AST levels with the progression of liver fibrosis, the abovementioned finding becomes logical.

Interestingly enough, our findings indicate that drugs used in ART, as well as both ART’s and each drug’s total duration are not associated with higher risk of liver fibrosis. Even though some ART drugs are accused for liver toxicity, especially those of the ‘first generation’ ART, this finding is not constant in studies made, since other ones, apart from our own, don’t associate type of ART to significant liver fibrosis [20, 22]. Moreover no HIV-related parameters, other than CD4/CD8 ratio, were correlated with liver fibrosis. The correlation of CD4/CD8 ratio with higher fibrosis risk is controversial. The CD4/CD8 ratio in normal individuals ranges from 1.5 to 2.5, and the low or inverted ratio is associated with altered immune function, immune senescence and chronic inflammation, as well as a variety of diseases, like neurocognitive disorders, lung cancer, and chronic obstructive pulmonary disease [30–37]. In HIV infected patients the chronic inflammation due to low CD4/CD8 ratio can possibly lead to chronic inflammation of the hepatocytes causing constant hepatocytes’ death and regeneration, which, in long term, could lead to liver fibrosis. Despite this, studies done in HCV/HIV co-infected and HIV mono-infected patients fail to agree in the involvement of low CD4/CD8 ratio in the liver damage of HIV-infected individuals [38–40].

The main limitations of our cross-sectional study are the small sample size of HIV/HCV and HIV/HBV co-infected, as well as the rather small size of the HIV mono-infected patients with severe fibrosis (equivalent to Metavir score of F3-F4). Finally, although liver biopsy is the gold standard for the assessment of liver fibrosis, no liver biopsies were used for detection and staging of liver fibrosis, because of the poor acceptability and the risk of possible adverse effects; however TE was used, a noninvasive method that has been found to be accurate and reproducible in determining the stage of liver fibrosis.

## Conclusions

In conclusion, in HIV-infected patients, HCV co-infection, older age, alcohol abuse and CD4/CD8 ratio alone or in combination, seem to correlate with fibrogenesis in the liver.

## Supplementary information

**Additional file 1.**

## Data Availability

The datasets used and/or analyzed during the current study are available from the corresponding author on reasonable request.

## Abbreviations

HIV: Human Immunodeficiency Virus
LS: Liver stiffness
TE: Transient Elastography
LREs: Liver Related Events
ART: Antiretroviral Treatment
HCV: Hepatitis C Virus
NRTIs: Nucleoside reverse transcriptase inhibitors
PIs: Protease Inhibitors
LFTs: Liver Function Tests
LB: Liver Biopsy
HDL: High Density Lipoproteins
LDL: Low Density Lipoproteins
TSH: Thyroid Stimulating Hormone
CDC: Centers for Disease Control and Prevention
ULN: Upper Limit of Normal
EBV: Epstein-Barr virus
HAV: Hepatitis A virus
HSV: Herpes simplex virus
VZV: Varicella zoster virus
AIDS: Acquired immune deficiency syndrome
KPa: Kilopascals
MSM: Men who have sex with men
IVDU: Intravenous drug users
AST: Aspartate Aminotransferase
ALT: Alanine Aminotransferase
γ-GT: Gamma-Glutamyl Transferase
ALP: Alkaline Phosphatase
HBV: Hepatitis B Virus
