# Supplementary material for: Causative factors of liver fibrosis in HIV-infected patients. A single center study
Source: BMC Gastroenterol. 2020 Apr 6;20:91. doi: 10.1186/s12876-020-01230-1 (PMC7137262; doi:10.1186/s12876-020-01230-1)
Supplement: Supplementary file 1 — Additional file 1. [file 12876_2020_1230_MOESM1_ESM.doc]

| **Drug category** | **Drug** | **Number of patients** | **Min duration** | **Max duration** | **Median duration** | **High vs Low fibrosis p value** |
| --- | --- | --- | --- | --- | --- | --- |
| **NRTI** | ZIDOVUDINE | 52 | 2 | 313 | 115.5 | 0.492 |
|  | DIDANOSINE | 24 | 6 | 199 | 97.5 | 0.866 |
|  | STAVUDINE | 10 | 7 | 195 | 54 | 0.756 |
|  | LAMIVUDINE | 86 | 1 | 282 | 102.5 | 0.786 |
|  | EMRICITABINE | 100 | 2 | 135 | 40.5 | 0.162 |
|  | TENOFOVIR DISOPROXIL FUMARATE | 106 | 2 | 154 | 52.5 | 0.086 |
|  | ZALCITABINE | 5 | 11 | 110 | 30 | 0.281 |
|  | TENOFOVIR ALAFENAMIDE | 2 | 6 | 6 | 6 | 0.686 |
|  | ABACAVIR | 46 | 1 | 128 | 24 | 0.115 |
|  | **Total in category** | **161** | **2** | **606** | **160** | **0.118** |
| **NNRTI** | EFAVIRENZ | 57 | 1 | 168 | 64 | 0.099 |
|  | RILPIVIRINE | 9 | 3 | 42 | 16 | 0.085 |
|  | ETRAVIRINE | 5 | 27 | 86 | 60 | 0.519 |
|  | NEVIRAPINE | 4 | 1 | 94 | 53 | 0.179 |
|  | **Total in category** | **68** | **1** | **192** | **61** | **0.093** |
| **P-INHIB** | LOPINAVIR | 45 | 4 | 196 | 82 | 0.374 |
|  | RITONAVIR | 81 | 4 | 282 | 72 | 0.086 |
|  | DARUNAVIR | 23 | 9 | 88 | 33 | 0.541 |
|  | INDINAVIR | 19 | 16 | 211 | 73 | 0.698 |
|  | SAQUINAVIR | 8 | 41 | 168 | 86 | 0.411 |
|  | FOSAMPRENAVIR | 5 | 24 | 118 | 88 | 0.519 |
|  | TIPRANAVIRE | 3 | 3 | 74 | 38.5 | 0.086 |
|  | ATAZANAVIR | 10 | 23 | 133 | 39.5 | 0.355 |
|  | **Total in category** | **90** | **8** | **450** | **134** | **0.126** |
| **INT-INHIB** | DOLUTEGRAVIR | 19 | 1 | 23 | 7 | 0.698 |
|  | ELVITEGRAVIR | 11 | 3 | 35 | 8 | 0.835 |
|  | RALTEGRAVIR | 35 | 1 | 112 | 27 | 0.659 |
|  | **Total in category** | **63** | **1** | **134** | **16** | **0.674** |
| **CCR5 inhibitor** | MARAVIROC | 4 | 12 | 60 | 45.5 | 0.565 |
| **HIV fusion inhibitor** | ENFUVIRTIDE | 3 | 30 | 76 | 74 | 0.619 |
| **Pharmacokinetic enhancer** | COBICISTAT | 12 | 1 | 35 | 8 | 0.908 |

**Appendix:** Total treatment exposure of the various drugs used by the patients along with the number of the patients that received each drug, minimum and maximum duration of treatment with the specific drug (in months), as well as median duration of treatment in months. The last column indicates p-values comparing the role of administration of each drug in patients with high vs. low fibrosis. Note that patients are treated by more than one drugs simultaneously and that each patient might have received more than one ART regimens due to t.ex. treatment failure or side effects.

NRTI: nucleoside reverse transcriptase inhibitor, NNRTI: non-nucleoside transciptase inhibitor, P-INHIB: protease inhibitor, INT-INHIB: integrase inhibitor, CCR5: C-C motif chemokine receptor 5, HIV: Human immunodeficiency virus
